# Evaluation of anti-proliferative activity of *Eryngium caucasicum* on melanoma cancer cells

**DOI:** 10.1186/s12906-022-03618-w

**Published:** 2022-05-14

**Authors:** Haleh Forouhandeh, Zahra Rezaei Param, Ommoleila Molavi, Parina Asgharian, Vahideh Tarhriz

**Affiliations:** 1 Molecular Medicine Research Center, Biomedicine Institute, Tabriz University of Medical Sciences, Tabriz, Iran; 2 Student Research Committee, Tabriz University of Medical Sciences, Tabriz, Iran; 3 Department of Pharmaceutical Biotechnology, Faculty of Pharmacy, Tabriz University of Medical Sciences, Tabriz, Iran; 4 Department of Pharmacognosy, Faculty of Pharmacy, Tabriz University of Medical Sciences, Tabriz, Iran; 5 Drug Applied research Center, Tabriz University of Medical Sciences, Tabriz, Iran

**Keywords:** Apoptosis, Cytotoxicity, *Eryngium caucasicum*, Melanoma

## Abstract

**Background:**

The genus Eryngium is a member of the Apiaceae family that has shown different pharmacological effects mainly including anti-inflammatory, analgesic, anti-cancer, hepatoprotective, and anti-oxidant. Previous research on the anti-cancer activity of Eryngium in some cancer cell lines has led us to explore the anti-proliferative activity of *E. caucasicum* in the B16F10 cell line.

**Objective:**

In this study, the antiproliferative activity of *E. caucasicum* on melanoma cancer cells (B16F10) and non-cancerous cells (HFFF2) were evaluated in vitro.

**Methods:**

The dried plant sample of *E. caucasicum* was extracted by Soxhlet apparatus with n-Hexane, dichloromethane, and methanol solvents. The effects of cytotoxicity of the extracts by the MTT method on melanoma cancer cells (B16F10) and noncancerous cells (HFFF2) was investigated for 24 and 48 hours. Then, the cytotoxicity of different fractions of the strong extract against normal and cancer cells was evaluated by this method. Annexin V/PI assay was used to study the induction apoptosis via the fractions in cancer cells.

**Findings:**

According to the results of the MTT test, n-Hexane extract is the most effective extract against the B16F10 cell line and is a candidate for fractionation with VLC. Among the fractions, 40 and 60% VLC fractions of n-Hexan extract inhibited the growth of B16F10 cells at 24 and 48 hours while, these fractions at IC_50_ concentration had no cytotoxic effects on normal cells. Treatment of cancer cells with effective extract caused apoptosis and necrosis and 40 and 60% more fractions induced apoptosis in these cells.

**Conclusion:**

The n-Hexane extract of *E. caucasicum* and its 40 and 60% fractions showed the highest cytotoxic effect against the B16F10 cell line compared to other extracts and control groups. This inhibition was made through induction of apoptosis.

## Introduction

Cancer mainly begins with the abnormal proliferation of cells that can invade and metastasize to other parts of the body [1, 2]. Cancer mortality is on the rise and has become one of the leading causes of human mortality [3]. Melanoma is a malignant neoplasm of melanin-producing cells (skin pigment) that is commonly found in the skin and sometimes in the mucosa and is the most dangerous common skin cancer [4]. Early diagnosis is an optimum opportunity for treatment. Systemic chemotherapy (the administration of drugs that kill cells throughout the body) and bio-chemotherapy (chemotherapy in combination with substances that can improve the immune response, known as immune cytokines, such as interleukin-2 and interferon-alpha) have been known mainstay to treat this cancer for more than three decades [5]. Previous studies have shown that herbal medicines can stop the growth and progression of tumors showing acceptable results without serious damage to healthy cells [6, 7]. Some compounds of natural origin such as Taxol (from *Taxus brevifolia*), Camptotechin (from *Camptotheca acuminate*), Vinca alkaloids (from *Catharanthus roseus* G.Don), and podophyllotoxin (from *Podophyllum peltuturn* L.) are currently used to treat various metastatic and non-metastatic malignancies. It is effectively used as an anti-cancer drug [8, 9]. Among the famous medicinal plants, the Eryngium genus from the Umbelliferae family has valuable compounds and is used in many cases indicating the effects, such as reduction of labor pain and placental abruption [10], treatment of fungal infections, hemorrhoids, and gastric ulcers [11, 12], lowering blood sugar [13], cytotoxic effects on cell lines Hep2, Hep G2, Vero, U138 MG [14], cytotoxic effects on melanoma cancer cells (A375), breast adenoma-carcinoma cells (MDA-MB231) and colon cancer cells (HCT116) [15], antioxidant effect [16], hepatoprotective [2], antipyretic, analgesic and anti-inflammatory effects [10, 17]. Considering the importance of medicinal plants to treat cancer and observe the cytotoxic effect of this species and other species of this genus on cancer cells, this species was selected to scrutinize its anti-cancer effect on melanoma cancer cells (B16F10).

## Materials and methods

### Preparation of plant samples

The shoots of ​ *E. caucasicum* plant was collected in June 2018 from the Namin region, Ardabil, Iran, (38°25′46.5″N 48°28′06.9″E). Ethical approval to collect ​ *E. caucasicum* was obtained from the Research Ethical Committee (REC) Number: IR.TBZMED.RDC.1398.108, Tabriz University of Medical Sciences, Tabriz, Iran. Dr. Fatemeh Ebrahimi (botanists) performed the taxonomic identification, and voucher specimen TBZ-FPH-1800 was deposited at the herbarium of Tabriz University of Medical Sciences, Tabriz, Iran for future reference.

### Preparation of extracts

The aerial parts of *E. caucasicum* were dried in the open air and then turned into a very fine powder by an electric mill. The powder obtained from this step is very light and has a low density. 200 g of the prepared powder was transferred to cartridge paper and placed in the Soxhlet machine. Extraction was performed using n-Hexane (n-Hex), dichloromethane (DCM), and methanol (MeOH) solvents for about 8 hours, respectively. Then, the obtained extract by rotary evaporator at 45 °C was dried under low air pressure and weighed.

### Preparation of n-hex extract fraction by VLC method

Inside the special funnel, VLC was covered with filter paper and filled with GF254 silica gel. By using a vacuum pump, we packed the silica gel and washed the column with a mixture of ethyl acetate and n-Hex, loading 2 g of the n-Hex extract in the column with 150 CC of a 10% ethyl acetate/ n-Hex solvent. Then, we washed the funnel with n-Hex using increasing percentages of ethyl acetate (10, 20, 40, 60, 80, and 100%), and each time, The fractions were collected separately using a rotary device. The extracts were completely dried by a rotary evaporator at 45 °C under low air pressure and after making sure that the extract is dried completely, it was dissolved in DMSO solvent. The amount of DMSO was less than 0.1%, which did not have a significant cytotoxic effect [18]. DMSO control was also included in the tests and cell viability was determined according to DMSO control [19].

### Cell lines used in experiments

Cell line B16F10 as a malignant melanoma cancer cell derived from male mice, and HFFF2 cell line as a normal foreskin fibroblasts cell derived from a human newborn, prepared by the Pasteur Institute, Iran.

### Cell cultures

The culture medium RPMI-1640 (Roswell Park Memorial Institute) was used as the general culture medium in this study. RPMI culture medium was purchased from Sigma Company in powder form. After adjusting the acidity, the culture medium was reduced to 1 l with deionized water and sterilized using 0.22 μm filters. 0.1 mg/mL streptomycin and 100 U/mL penicillin G were added to the medium. The prepared medium was kept in the refrigerator at 4 °C and enriched with 10% of *Fetal Bovine Serum* (FBS). All preparation steps of culture medium were performed under aseptic conditions.

### Evaluation of cytotoxicity of extracts by MTT method

B16F10 and HFFF2 cells were cultured separately in a flask, and after proper cell growth, the cells were separated from the flask. 200 μl of cell suspension was taken into each of the 96-well plate containing 15× 1000 cells/well. The cells were incubated for 24 hours at 37 °C, 5% CO_2_ and 95% humidity. In the next step, the cells were treated in triplicate with n-Hex, DCM, and MeOH extracts of *E. caucasicum* at zero concentrations (containing only DMSO), 1, 15, 30, 50, and 80 μg/ml. The test was carried out in two series, in the first series, the plates were incubated for 24 hours and the incubation of plates was taken for 48 hours in the second series. After the treatment period, the plates were removed from the incubator. Then, the morphology of the cells was examined for several minutes through the inverted light microscope. Afterward, the culture medium of the wells was drained and the cells were washed using PBS, and 150 μl of the prepared MTT solution was added to each well. The absorption of the plates was measured by ELISA Plate Readers at 570 nm [20]. After determining the data of the first stage and the relevant statistical analyzes, stronger extracts were selected and fractionated for further studies. The effective extract fractions were again examined by MTT assay to study cytotoxicity [21]. Concentrations of fractions in cell treatment were zero, 1, 15, 30, 50, 80 and 100 μg / ml at 24 and 48 hours.

### Detection by flow cytometry

During apoptosis, phosphatidylserine is transported from the inner surface to the outer surface of the membrane, with the annexin V marker attached to the extracellular phosphatidylserine and the PI marker attached to the fragmented DNA of the dead cell nucleus, detectable by Flow cytometry. To start the flow cytometry test, the first 2 × 10^4^ cells were distributed in each 6-cell well plate. After washing the cells with PBS, 150 μl of trypsin was added and diluted with 50 μl of PBS. The cell density was 2–5 × 10^4^ cells per milliliter per microtube. 5 μl of Annexin V and 5 μl of Propidium Iodide were added to each microtube except for unstained control. They were located in a dark place at room temperature for 15 minutes. After the incubation time, the microtubes were centrifuged and after draining the supernatant, 100 μl of the diluted Annexin V buffer was added and the adsorption was read by Flow cytometry. For each cell line, two volts are related to uncolored control, and two volts are related to colored control, all 4 volumes do not contain active plant material. Plant samples were treated at IC_50_ concentrations and after 24 hours of incubation, Flow cytometry was performed by the discussed method [22, 23]. Non-color control was used to remove any background adsorption and color control was used to prevent the involvement of apoptosis or normal cell necrosis in the results of the samples.

### GC/MS analysis

1 μL of the n-Hex extract and its fractions were injected into the apparatus of Shimadzu GCMS-QP5050A equipped with a DB-1 column (60 m × 0.25 mm; film thickness 0.25 μm). The initial oven temperature was 50 °C for 3 minutes that raised to 270 °C at a rate of 4 °C/min finally the injection temperature was set up at 240 °C according to the described method by [24, 25].

### Statistical analysis

The results of UV absorption at 570 nm from the Plate reader with two replications were entered into GraphPad Prism6 Demo 8 software. The software automatically determined the mean ± SD, plots the graph and calculates the IC_50_ for each experiment. ANOVA and Tukey post hoc test was also used to assess the *p*-value.

## Results

### Amounts of extracts and fractions

The extracts were obtained from 200 g of aerial part powder of *E. caucasicum* through Soxhlet method and solvents of n-Hex, DCM, and MeOH are shown in Table 1. After cytotoxicity tests, as the n-Hex extract was stronger than the other two extracts, therefore the fractions were prepared from this extract. The results of fractionation of 2 g of n-Hex extract by VLC method are also shown in Table 1.

**Table 1:** Results were obtained from weighing n-Hex, DCM, and MeOH extracts and fractions from 200 g of *E. caucasicum* aerial powder

MeOH		DCM		n-Hex		Extract
3.18		2.12		3.68		Weight of plant extract (g/100 g of extract)
**100%**	**80%**	**60%**	**40%**	**20%**	**10%**	Fractions
6.7	10.35	12.05	13.01	5.5	1.1	Weight of n-Hexane extract fractions (g/100 g of extract)

### Investigation of cytotoxic effects

#### Investigation of cytotoxic effects of the studied plant by MTT assay

**Fig. 1:**
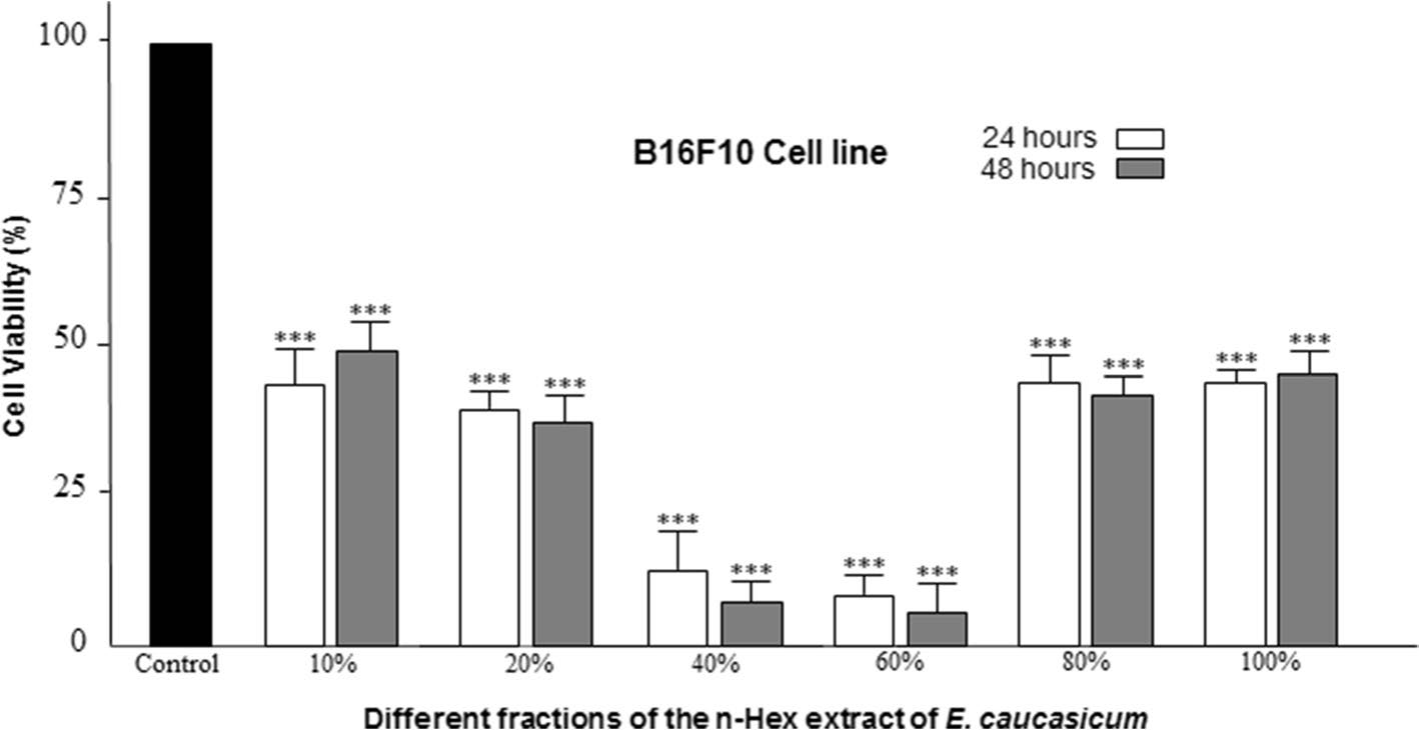
Viability of B16F10 cancer cells after exposure to incremental concentrations of different fractions of the n-Hex extract of *E. caucasicum* using MTT method after 24 and 48 hours of incubation. *p < 0.05, **p < 0.01, and ***p < 0.001 indicate comparison treated groups with non-treated (Control) group

One of the standard methods to assess the level of cytotoxicity is to determine IC_50_, which is the concentration of the drug that kills 50% of the cells. The IC_50_ values ​​from the GraphPad Prism 8.0.1 software are shown in Table 2. According to the table, low IC_50_ values ​​of n-Hex extract in the tested cell line (B16F10) indicate high cytotoxicity of this extract. Therefore, hexane extract is a candidate for fractionation with VLC. The results of cytotoxicity of n-Hex extract on B16F10 cells are shown in Fig. 1. To evaluate the selective cytotoxicity of strong extracts and their fractions, the HFFF2 cell line, MTT test was performed on the mentioned cell line, which its results are shown in Table 3.

**Table 2:** IC_50_ values of extracts and fractions of the most effective extract on cancer cells (B16F10) after 24 and 48 hours in μg / mL

IC_50_ (μg/ml)									
**Cell line**	**Extracts**			**VLC fractions of n-Hex**					
	n-Hex	DCM	MeOH	10% VlC fraction	20% VlC fraction	40% VlC fraction	60% VlC fraction	80% VlC fraction	100% VlC fraction
B16F10 24 h	**14.55 ± 1.3**	31.22 ± 2.7	47.59 ± 6.6	31.33 ± 20.7	23.87 ± 18.4	**8.25 ± 3.03**	**5.68 ± 0.59**	45.81 ± 28.2	39.83 ± 21.6
B16F10 48 h	**11.03 ± 1.65**	24.77 ± 0.36	36.37 ± 5.7	48.57 ± 3.1	30.41 ± 4.1	**5.71 ± 0.4**	**4.68 ± 0.08**	33.41 ± 4.1	35.99 ± 3.8

**Table 3:** IC_50_ values of extracts and strong fractions on normal cells (HFFF2) after 24 hours in μg / mL

IC_50_ (μg/ml)			
Cell line	n-Hex extract	40% VlC fraction of n-Hex extract	60% VlC fraction of n-Hex extract
HFFF2	55.39 ± 0.28	19.79 ± 3.45	16.02 ± 5.2

#### Significant comparison of the effect of different samples with controls and detection of major compounds

**Fig. 2:**
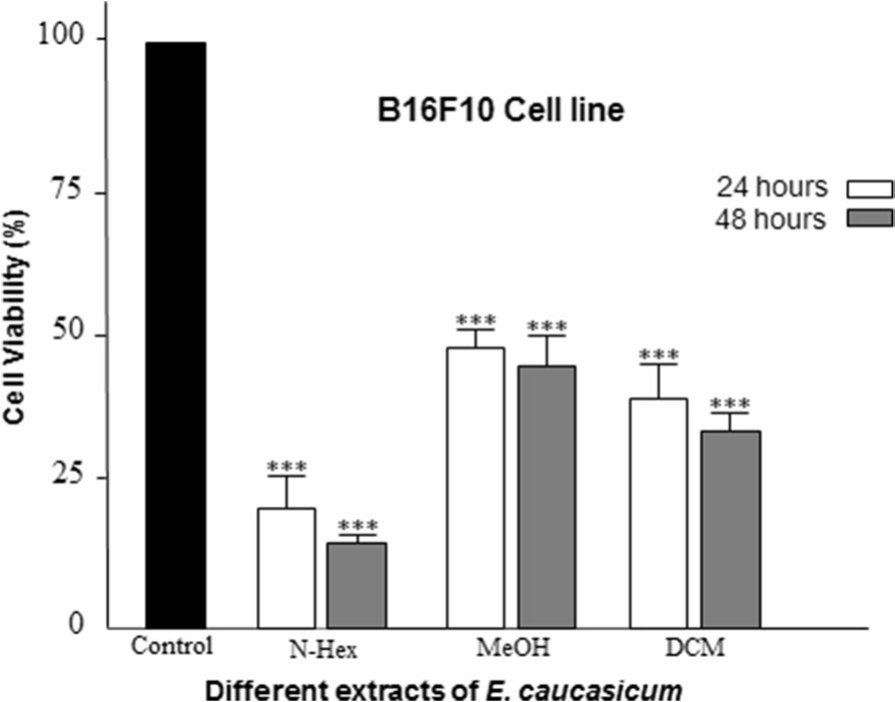
Statistical comparison of B16F10 cell viability due to exposure to three types of n-Hex, MeOH, and DCM extracts after 24 and 48 hours of incubation compared with DMSO control group. *p < 0.05, **p < 0.01, and ***p < 0.001 indicate comparison treated groups with non-treated (Control) group

**Fig. 3:**
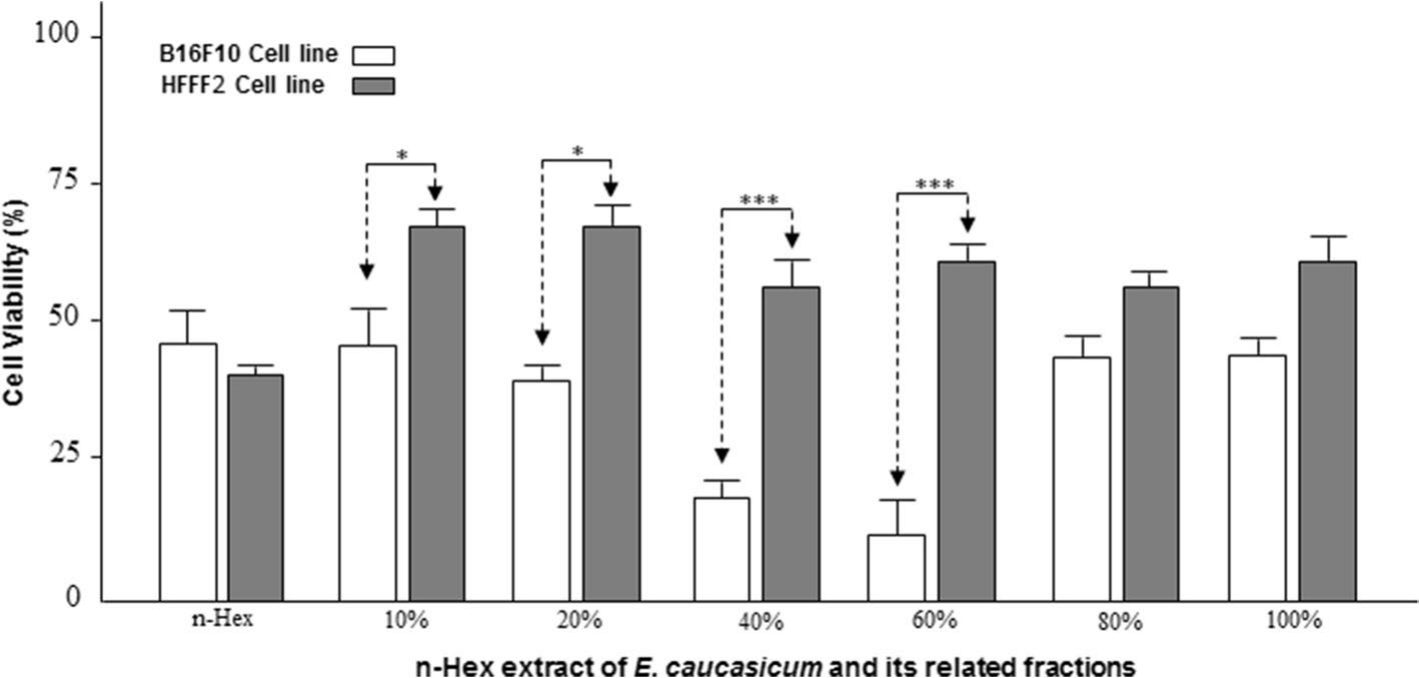
Viability of B16F10 cancer cells due to exposure to potent extract (n-Hex) and its fractions of *E. caucasicum* compared to normal HFFF2 cells − 2 in 24 hours. *p < 0.05, **p < 0.01, and ***p < 0.001 indicate comparison treated groups with control group

To compare the cytotoxic effects of different plant samples at 24 and 48 hours, Two Way ANOVA and TUKEY post hoc test were used. In statistical tests, the significant level was expressed as *** (*p* < 0.001), ** (*p* < 0.01), * (*p* < 0.05) ns (*p* > 0.05). The viability of B16F10 cells after treatment with 3 types of extracts and fractions of n-Hex extract after 24 and 48 hours of incubation is shown in the following diagrams. Statistical analysis of ANOVA and Tukey post hoc test showed that the extracts and their fractions were significantly different from the DMSO control group (Fig. 2) at both incubation times (24 and 48 hours). Figure 3 illustrates the viability of B16F10 and HFFF2 cells in the face of stronger extracts and fractions (n-Hex extract and 40 and 60% fractions of the said extract) in 24 hours. The rate of reduction of B16F10 cells compared to HFFF2 cells in the presence of both fractions was significant. However, this was not the case with the extract. Based on the GC/MS analysis, the main compounds of n-Hex extract were trimethylbicyclo [2.2.1] octane-2-ol (10%), tetradecane (6.3%) and hexadecanoic acid (9.8%). In addition, the effective compounds in 40 and 60% fractions of n-Hex extract were detected as 9-octadecanoic acid (3.24%) and hexadecanoic acid-2hydroxy- methyl ester (4.56%), respectively.

#### Flow cytometry test results on B16F10 cells

**Fig. 4:**
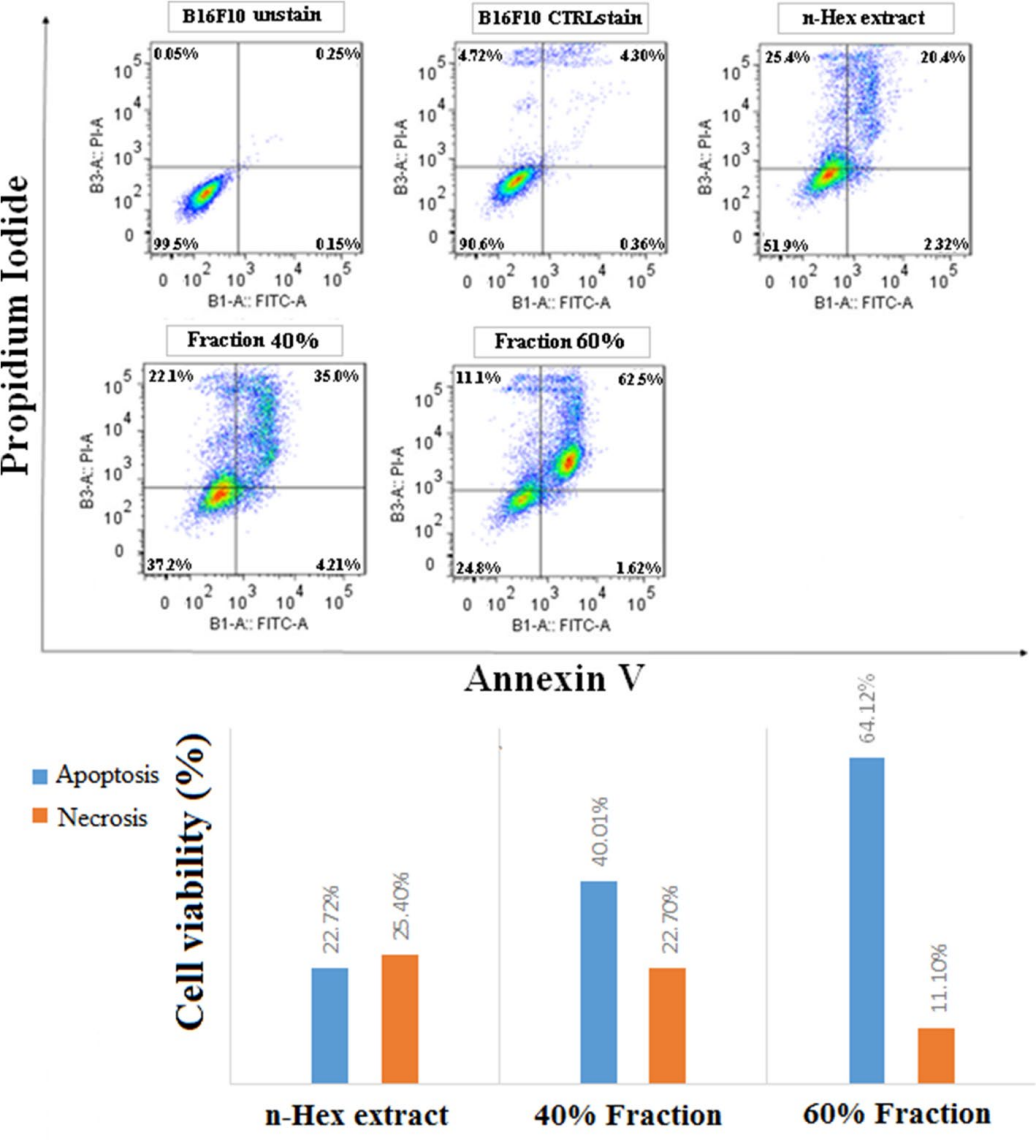
Flow cytometric test of effective extract and its potent fractions against B16F10 cell line and comparison of apoptotic and necrotic cells in B16F10 cell line

**Fig. 5:**
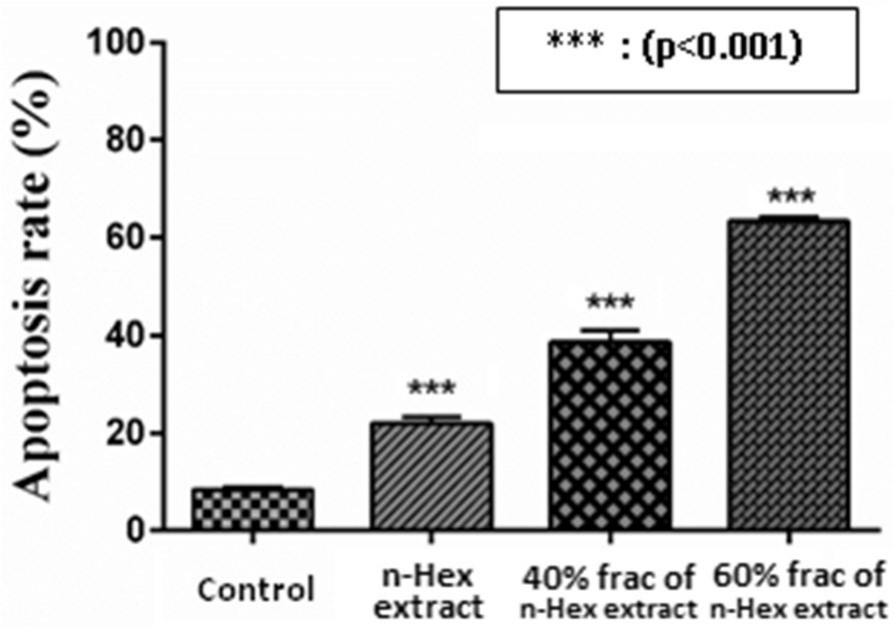
Comparison of apoptosis rates in B16F10 cell line after 24 hours

During apoptosis, changes occurred in the cell membrane, which could be detected by a Flow cytometry test. In the early stages of apoptosis, phosphatidylserine was transferred from the cytosolic side to the outside of the membrane, binding to annexin V in this test. The end of apoptosis and necrosis of the PI marker was also attached to the fragmented DNA of the nucleus of dead cells. Thus, the percentage of annexin V positive indicated the extent of cell progression to apoptosis. In this test, potent extract (n-Hex) and potent fractions (40 and 60% n-Hex extract) of *E. caucasicum* were incubated with B16F10 cancer cells for 24 hours at IC_50_ concentration. The histogram of apoptosis induction and the graph of apoptotic and necrotic cells are presented in Figs. 4 and  5.

## Discussion

According to the World Health Organization, 9.6 million deaths in 2018 worldwide were due to various types of cancer, and it is predicted that the number of these deaths will reach more than 11 million people per year in 2030 (https://www.who.int/en/news-room/fact-sheets/detail/cancer). Malignant melanoma is the most dangerous common skin cancer. The prevalence of malignant melanoma is significantly increased in areas such as Canada and Australia that are exposed to strong sunlight [5, 26]. Melanoma B16F10 cells are highly metastatic. Cell invasion, migration, and adhesion behaviors are important features of cancer metastasis. Many studies have shown that inhibition of these stages leads to the prevention of metastasis and are the development goals of anti-cancer agents. The data showed that active n-Hex extracts such as macluraxanthone and gerontoxanthone-I significantly inhibit both B16F10 cell invasion and regeneration of matrix/fibronectin regenerated membranes in the transwell chamber in a dose-dependent manner [27].

Treatments such as surgery, chemotherapy, and radiotherapy are usually the first clinical options for most cancer patients, but these treatments are not remedial in many patients and have limitations including treatment costs, inefficiencies in advanced tumors, and side effects. They are abundant in the treatment of patients [28]. Considering the side effects of chemotherapy drugs, many studies have been done to reduce these side effects. Many synthesized anticancer drugs, including taxanes, vinca alkaloids, podophyllotoxins, and comptotsins, are derived from plant compounds and are used to treat various metastatic and non-metastatic cancers [29]. Studies have shown that plant compounds play an important role in cancer prevention and treatment. These compounds act by different mechanisms, but the induction of apoptosis is a common point of many of these compounds [30]. In the present study, the cytotoxic effects of *E. caucasicum* on cancer and non-cancer cells were investigated. For this purpose, B16F10 cells were used as cancer cell lines and HFFF2 cells were used as normal cell lines. In this regard, after culturing, cytotoxicity and IC_50_ values ​​were obtained from the treatment of these cells with different extracts of n-Hex, DCM, and MeOH. The plant was evaluated at different concentrations and times (24 and 48 hours). Then the n-Hex extract as the most effective extract was fractionalized by using the VLC method and the cytotoxicity effects of its fractions were investigated. All statistical calculations were significant at the *p*-value. Thus, the mechanism of cytotoxicity of the extract and the effective fractions were investigated by Flow cytometry annexin V/PI kit. Statistical analysis of ANOVA and Tukey post hoc test showed that all three types of extracts including n-Hex, DCM, and MeOH, in both incubation times (24 and 48 hours) in the B16F10 cancer cell line, were significantly different (*p* < 0.001) from the DMSO control group. The IC_50_ content of n-Hex extract of *E. caucasicum* on B16F10 was lower than that of DCM and MeOH extracts. This extract is dose-dependent and reduces the viability of cells by increasing the concentration of plant samples. The mechanism of its toxicity is apoptosis and necrosis. Also, the effect of cytotoxicity of the drug increases over time (*P* = 0.0057). Since n-Hex extract had the highest cytotoxic effect on cancer cells, fractionation with VLC was performed. In addition, our finding showed that the fractions of n-Hex extract in cancer and normal cell lines at both incubation times (24 and 48 hours) had a significant difference (*p* < 0.001) with the DMSO control group with concentration-dependent effects, except for the 10% fraction. Moreover, in cell line B16F10, the viability of cells in the face of 60 and 40% fractions with the lowest IC_50_ values are significantly different (*p* < 0.001) from other fractions and, as mentioned earlier, have dose-dependent but independent of time. Flow cytometry test, indicated the amount of apoptosis. After performing the test on potent extract (n-Hex), it was found that it inhibits the growth of cancer cells through both apoptosis and necrosis; also, the resulting potent fractions (40 and 60% fractions) act mostly through apoptosis. It is noteworthy that the cytotoxic compounds in the 60% fraction led to 64.12% of the treated cells leading to apoptosis, which has the highest rate of apoptosis among the studied samples. Based on the results, it can be concluded that the apoptosis-inducing compounds are probably in the n-Hex extract and a larger amount of them are presented in the 60% fraction. The 60% fraction due to the appropriate cytotoxicity, concentration dependence (*p* < 0.0001), being independent of time and high induction percentage of apoptosis in cancer cells. The analysis of 40 and 60% fractions of n-Hex extract compounds showed that the effective compounds were 9-octadecanoic acid (3.24%) for 40% fraction and hexadecanoic acid-2hydroxy- methyl ester (4.56%) for 60% fraction. The separation and purification of regarded compounds can be promising options for future clinical research. However, the investigation of chemical analyzing of other fractions and their comparison is highly recommended in the next phytochemical field in future studies. Phytochemical studies on the genus Eryngium have shown that it contains primary phenolic compounds and terpenoids, including triterpene saponins as the main compound, monoterpene, sesquiterpene, triterpenoids, flavonoids, coumarin, steroids, acetylene, and other compounds [31]. In general, compounds in the genus Eryngium such as essential oil, sterol, and saponin are responsible for anti-cell growth activity [32]. Hydrocynamic derivatives such as caffeic acid, chlorogenic acid, synaptic acid, ferulic acid, and para-coumaric acid, found in the genus Eryngium, have antioxidant properties and inhibit cancer growth [33, 34]. Previous research has shown that different species of the genus Eryngium have cytotoxic effects on different cell lines such as MCF-7, PANC-1, HT-29, PC-3, HL-60, HepG-2, and A-5749 [35, 36]. Kartal et al. indicated that three eryngiosides of the genus Eryngium (eryngioside J, eryngioside L and saniculasapoinin III) had moderate cytotoxic properties against PANC-1, A-549, PC-3, HL-60 and MRC-5. These eryngiosides were more effective on A-549 and MRC-5 and significantly inhibited the growth of PANC-1 cells. However, isolates of *E. campestre* root have shown less toxicity against HCT-116 and HT-29 [37]. A study found that *E. creticum* contains alkaloids, tannins, coumarins, saponins, flavonoids, and polyphenols that play a key role in cytotoxicity and apoptosis. Furthermore, the ethanolic and aqueous extracts of the leaves of this plant have the greatest effect on cytotoxicity on the HeLa cell line for 48 hours [38, 39]. *E. capmpestre* essential oil on cell lines A-375, MDA-MB-231 and HCT-116 has appropriate cytotoxicity, which is comparable to cisplatin chemotherapy [40]. There are also studies on the anti-cell growth effect of *E. creticum* on MCF-7. Aqueous and ethyl acetate extracts of the leaves and stems of the plant did not have a significant toxic effect on MCF-7, while methanolic extract with a lower IC_50_ caused cell toxicity [41]. The shoots and roots of *E. maritimum* and *E. kotschyi* induced cytotoxicity in U-138-MG, HepG2 [14]. Ethanolic extract of *E. planum* fruit induced the most apoptosis in 24 hours in leukemia C8166 and J-45 cancer lines by 96 and 89%, respectively [42]. It has been shown that the whole MeOH extract from the roots, flowers, and leaves of *E. serbicum* had selective anti-cell growth effects on HCT-116, SW-480, MDA-MB-231, and normal MRC-5 cancer cells. Flow cytometry results also revealed that these extracts induce apoptosis. Phytochemical studies demonstrated that chlorogenic and rosmarinic acid is the predominant compounds in this plant that may be responsible for cytotoxicity [43]. n-Hex extracts of different species of Eryngium have shown good cytotoxic and antioxidant effects in animal models, which can be due to the reduction of nitric oxide and TNFα [17, 44]. The effects of n-Hex extract of *E. billardieri* on the PANC-1 cell line induce cytotoxicity and apoptosis by increasing Bax gene expression and decreasing cyclin D1 gene expression without affecting normal cells [45]. In terms of mechanisms of action, morphological changes of apoptotic cells such as membrane bubbles, chromatin density, nucleus fragmentation, apoptotic bodies, and loss of adhesion, as well as cell cycle arrest in the G1 phase were observed by increasing the proportion of cells sub-G1. These mechanisms are detected by Hoechst 33342 staining, and flow cytometry showed DNA damage and subsequent apoptotic cell death [27]. Sarcodiol (SD) inhibits the expression levels of signal transducers and transcription protein activators (STAT-3) and cyclin D1, a cyclin-dependent kinase 4 activator (Cdk4). SD treatment increases the cellular level of the tumor suppressor protein 53 (p53) and stimulates nuclear polymerase (ADP-ribose) cleavage. SD treatment also increases cleaved cell levels of caspase-3, − 8, − 9 and stimulates the enzymatic activities of caspase-3, − 8, and − 9. These results, in addition to inhibiting cell viability show that SD prevents melanoma cell proliferation by stopping the cell division cycle in the silent phase and promotes programmed cell death (apoptosis) through active external and internal pathways [46].

## Conclusion

In the present study, the main aim is the investigation of anti-cancer effect of *E. caucasicum* extracts and detection of major effective compounds. Therefore, the different extracts of plant were evaluated on B16F10 melanoma cancer cell line compared to untreated B16F10 cells. Our finding showed that the n-Hex extract of *E. caucasicum* and its 40 and 60% fractions have significant cytotoxicity on melanoma cancer cells compared to untreated B16F10 cells. In next step, to detect whether these are selective on cancer cells or not, we prepared same tests on HFFF2 as a control cells. Our results confirmed that 60 and 40% fractions of n-Hex extract have superior cytotoxic impact on cancer cells compared to control cells. It is worth mentioning that the mechanism of action of n-Hex extract is in the form of apoptosis and necrosis; however, in the case of these fractions, it is mainly through induction of apoptosis. On the other hand, these fractions have selective toxicity effects on cancer cells, and inhibition of cell growth is time-independent. The analysis of 40 and 60% fractions of n-Hex extract compounds showed that the effective compounds were 9-octadecanoic acid (3.24%) for 40% fraction and hexadecanoic acid-2hydroxy- methyl ester (4.56%) for 60% fraction. Therefore, 60 and 40% fractions of n-Hex extract can be candidate for future studies as anti-cancer compounds by separation of their pure cytotoxic compounds.

## Data Availability

The datasets used and/or analyzed during the current study are available from the corresponding author on reasonable request.
